# Health and related behaviours of fly-in fly-out workers in the mining industry in Australia: a cross-sectional study

**DOI:** 10.1007/s00420-022-01908-x

**Published:** 2022-07-25

**Authors:** Bernard Yeboah-Asiamah Asare, Suzanne Robinson, Daniel Powell, Dominika Kwasnicka

**Affiliations:** 1 Curtin School of Population Health, Curtin University, Kent Street, Perth, WA 6102 Australia; 2 Health Psychology, Institute of Applied Health Sciences, University of Aberdeen, Aberdeen, UK; 3 Deakin Health Economics, Faculty of Health, Deakin University, Burwood, Australia; 4 Rowett Institute, University of Aberdeen, Aberdeen, UK; 5 Faculty of Psychology, SWPS University of Social Sciences and Humanities, Wroclaw, Poland; 6 NHMRC CRE in Digital Technology to Transform Chronic Disease Outcomes, Melbourne School of Population and Global Health, University of Melbourne, Melbourne, Australia

**Keywords:** Psychological distress, Physical health, FIFO, Mining, Health behaviours, Australia

## Abstract

**Background:**

Fly-In Fly-Out (FIFO), which entails travelling mostly from the urban areas to stay and work in remote areas for designated periods and travel back home to spend designated days of leave, has become a common work arrangement in the mining sector globally. This study examined the mental and physical health of FIFO workers and described their health-related behaviours during on-and off-shift periods.

**Methods:**

A cross-sectional study was conducted with FIFO workers (*N* = 216) in the mining industry in Australia who completed an online survey. Paired *t*-test and McNemer’s analysis examined the differences in health-related behaviours during workers’ on-and off-shift days. Logistic regression examined the predictors of physical health and psychological distress status of FIFO workers.

**Results:**

Workers reported longer sleep duration (7.5 ± 1.5 h vs 6.3 ± 1.2 h, *p* < 0.001) and better sleep quality (78.2% vs 46.3%, *p* < 0.001) during off-shift nights than on on-shift nights. Smoking prevalence was 26.4%, and workers reported smoking a similar number of cigarettes per day during on-and off-shift days. Most workers reported drinking alcohol (86.1%) and more often at risky levels during off-shift than on-shift days (57.9% vs 34.3%, *p* < 0.001). Fruits and vegetable consumption was low but with higher vegetable intake during off-shift days (2.8 ± 1.4 vs 2.3 ± 1.3 serves, *p* < 0.001). Workers had good physical health status (91.2%), but 71.4% were overweight/obese and 33.4% indicated high levels of psychological distress. Working on long shifts (OR 6.63, 95% CI 1.84–23.91) and smoking (OR 7.17, 95% CI 2.67–19.26) were linked to high psychological distress.

**Conclusions:**

The prevalence of psychological distress and risky health behaviours was high. Interventions should aim to reduce psychological distress and support multiple behaviour changes, considering FIFO work-related characteristics including long shift hours.

## Introduction

Rotation work arrangements, which entail travelling mostly from the urban areas to stay and work in remote areas for designated periods and travel back home to spend designated days of leave (Storey 2001, 2010), have in recent times become common in the mining sectors (Storey 2010). Originally designed to staff the remote offshore oil and gas fields (Storey 2001), rotation work arrangements, frequently denoted as *Fly-in Fly-out (FIFO)*, have come to be a standard practice in the onshore mining industry worldwide, particularly in Australia (Storey 2001), where the mining operations usually take place in rural and remote areas (Storey 2010).

Typically, FIFO jobs are associated with comparatively high earnings and lengthy periods of time off work to spend with families (Henry et al. 2013; Storey 2016). But other features of FIFO work, including recurrent separations from families for a period, long and compressed roster and shift patterns, and increased workloads (Vojnovic et al. 2014a), are deemed stressors that could impact negatively on the health and well-being of workers (Asare et al. 2021a; Vojnovic et al. 2014a). Workers experience isolation and loneliness (Barclay et al. 2016; Gardner et al. 2018), inability to meet the demands at home when away (Ebert and Strehlow 2017; Gardner et al. 2018) and family and social relationships disruptions (Gardner et al. 2018; Torkington et al. 2011). Workers are also presented with two (work and home) lives which come with different ways of living, roles and tasks, requiring the assumption of distinct social roles and behavioural patterns (Gardner et al. 2018).

According to the work-family theory (Greenhaus and Beutell 1985), interference between the discharge of work and family roles arising from the demands of these roles could result in stress-related problems, such as misuse of substances and mental distress (Allen et al. 2000), at levels dependent on the significance of the unaccomplished task (Greenhaus and Beutell 1985). Again, the Job Demands-Resources (JD-R) Model (Bakker and Demerouti 2007) stipulates that high job demands contribute to strain, including psychological distress.

Mental health problems and suicide concerns (Henry et al. 2013; Vojnovic et al. 2014b), and risky health behaviours, such as risky alcohol consumption (Vojnovic et al. 2014b), have been highlighted among FIFO workers in Australia. Studies have reported higher levels of psychological distress in FIFO workers than in the general population in Australia (Bowers et al. 2018; James et al. 2018). High rates of depression (Miller et al. 2019; Vojnovic and Bahn 2015), anxiety (Vojnovic and Bahn 2015), stress (Vojnovic and Bahn 2015) and suicide (Miller et al. 2019) have also been found among FIFO workers in Australia. However, inconsistent observations have been made on mental health in FIFO workers (Asare et al. 2021a); with other studies reporting a lower prevalence of mental health problems than found in other workgroups in Australia (Joyce et al. 2013), and statistically similar levels of depression and anxiety in FIFO workers and non-FIFO populations in Australia (Cooke et al. 2019; Dittman et al. 2016). Another study has found higher levels of depression and suicide risk among residential/non-FIFO mining workers than in FIFO workers (Miller et al. 2019).

Additionally, studies have also reported sleep problems such as short sleep duration and poor quality of sleep (Ferguson et al. 2010; Paech et al. 2010), high prevalence of overweight and obesity (Barclay et al. 2016; Joyce et al. 2013), higher alcohol consumption, and smoking rates (Joyce et al. 2013) among FIFO workers compared to other workgroups (Joyce et al. 2013) and the normal population (Dittman et al. 2016) in Australia. A high proportion of FIFO workers engage in insufficient physical activity in Australia (Clifford 2009; Joyce et al. 2013). In contrast, other studies have reported moderate alcohol consumption (Muller et al. 2008) and low smoking rates (Barclay et al. 2016) among FIFO workers and documented no significant differences in engaging in physical exercise and consuming fruits and vegetables between FIFO workers and workers in other employment types in Australia (Joyce et al. 2013).

FIFO work-related characteristics are associated with health issues (Vojnovic et al. 2014b). For instance, studies have demonstrated shift length (Bowers et al. 2018; James et al. 2018), e.g., working shifts of more than 12 h (James et al. 2018), roster patterns, e.g., 2 weeks on/1 week off (Bowers et al. 2018) and day/night shift rotation (James et al. 2020) as predictors of high psychological distress among FIFO workers in Australia. Shift patterns (day and night shift) (Asare et al. 2021a, b; Parkes 2012) and working longer shift hours (Parkes 2015) have also been identified to be linked to reduced sleep duration and poorer sleep quality in FIFO workers. Other studies have also found FIFO/rotation job roles/types as predictors of physical health complaints such as musculoskeletal pains (Bjerkan 2011).

A recent review examining the health and well-being of FIFO workers reported inconsistent findings regarding the impact of FIFO work on mental health outcomes, as well as very few studies examining its impact on health-related behaviours and physical health outcomes (Asare et al. 2021a). There are also limited studies of work-related factors that may be associated with the health and well-being of FIFO workers (Asare et al. 2021a). More research is suggested to examine the physical and mental health needs of FIFO workers and examine the work-related factors associated with their health outcomes (Asare et al. 2021a). Understanding the health status, lifestyle behaviours during on-and off-shift days and factors that contribute to the health and well-being of FIFO workers is critical for developing interventions to support and improve their health and well-being. The study aimed to examine the lifestyle behaviours of FIFO workers during on-and off-shift days. Second, the study aims to assess the self-reported mental and physical health and identify the FIFO work-related determinants of mental and physical health outcomes.

## Materials and methods

### Study design and participants

A cross-sectional study was carried out among mining workers aged 18 years and above who worked on FIFO schedules in Australia. The sample size needed to detect a small effect size (*d* = 0.20) difference between on-and off-shift days in a paired samples t-test, with 80% power, was 199 participants. Second, we also wanted to estimate the likely prevalence of health outcomes: using psychological distress as an example, we assumed at least 21% of our study participants would experience distress—as the midpoint of estimates from previous studies of FIFO mining workers suggesting prevalence ranging from 10% to 31.6% (Barclay et al. 2016; Bowers et al. 2018; James et al. 2018; Lester et al. 2015; Sellenger and Oosthuizen 2017). Using the Cochran’s sample size formula; *n* = *Z*^*2*^*p(1-p)/e*^*2*^ to capture at least 21% (*p*) of psychological distress, with 95% confidence interval (*z* = 1.96), and 5% margin of error (*e*), we needed 255 participants. Using this larger number, and assuming a 10% dropout rate, we aimed to recruit 280 participants. Data collection was conducted from July to December 2021. During that period, 326 FIFO workers were recruited through a non-probability convenient sampling technique, of which 299 (giving a participation rate of 91.7%) consented to take part in the study.

### Study procedure

Recruitment of study participants was done in two ways; first, a large mining company, with multiple mine sites and an estimated FIFO or Drive-in Drive-out (DIDO) work population of 2600 in Western Australia was approached for consent and the advertising materials of the study were sent through the company’s intranet weekly communications to invite their workers. Second, the advertising materials of the study were also posted in FIFO work support groups on the social media platform Facebook to recruit general FIFO mining workers in Australia. FIFO workers interested in taking part in the study were directed to use a URL link or QR code to access the online participants’ information and consent form. The participants completed an online questionnaire, administered through the Qualtrics XM online survey software (https://www.qualtrics.com/au/). All the participants confirmed that they worked in FIFO work in the mining industry. There was no mechanism to determine the proportion of study participants that were recruited through the mining company and/or from Facebook posts.

### Data collection tool and outcome measures

The study included the following outcome variables: psychological distress, physical health, alcohol use, smoking, physical activity, weight/obesity and fruits and vegetable intake, and the measures are described in detail below.

### Psychological distress

The Kessler Psychological Distress Scale (K10) (Kessler et al. 2002) was employed to measure the current level of psychological distress of FIFO workers. The scale, consisting of 10 items, measures the negative emotional states in the last 30 days (e.g., *In the last 30 days how often did you feel….nervous, depressed, hopeless, restless or fidgety*). The responses were rated on a 1 *(none of the time)* to 5 *(all of the time)* Likert scale. The K10 scale has been validated, with an internal consistency high (α = 0.93) (Kessler et al. 2002), and its sensitivity has been established in the Australian population (Furukawa et al. 2003). With the possible scores of 10–50, the psychological distress of participants was characterised as *low (10–15), moderate (16–21), high (22–29) and very high (30–50) levels*.

### Physical health

Physical health status was measured using the Physical Component Summary (PCS) of the SF-8 Health Questionnaire (Ware et al. 2001). The SF-8 has 8 items measuring the quality of life in the last 4 weeks, with a PCS subscale. The PCS subscale has 4 items (e.g., *How much bodily pain have you had during the past 4 weeks?*) and scored on a 5 and 6-point Likert scale. The scores were transformed to generate a total score ranging from 0 to 100 as per the SF-8 scale, with a higher score suggestive of better physical quality of life (Ware et al. 2001). The test–retest reliability for the subscale PCS-4 has been demonstrated as adequate at 0.73.

### Sleep and health-related behaviours

#### Sleep measures

Participants recalled their typical sleep duration and sleep quality for both on-and off-shift days using single items adopted from the *Pittsburgh Sleep Quality Index (PSQI)* (Buysse et al. 1989). Sleep duration was assessed by the item *“How many hours of actual sleep did you get at night during on (or off)-shift days?”* and sleep quality with *“During on (or off)-shift days, how would you rate your sleep quality overall?”* scored on a 4-point Likert scale from 0 (very good) to 3 (very bad) (Buysse et al. 1989). As indicated, separate questions were asked for on-and off-shift days. Single items were chosen for brevity and the use of single items for sleep duration and sleep quality is consistent with a previous study (Parkes 2015).

#### Alcohol intake

The current alcohol use and related behaviours in the last 1 year were assessed for on-and off-shift days with the *Alcohol Use Disorders Identification Test-Concise (AUDIT-C)* (Bradley et al. 2007). AUDIT-C is a brief validated tool consisting of 3 items: “*How often do you have a drink containing alcohol?”;* “*How many standard drinks containing alcohol do you have on a typical day when drinking?”;* “*How often do you have six or more standard drinks on one occasion?”* Each item was scored on a 5-point scale (0 to 4) for screening for risky alcohol consumption. Separate questions were asked for on-shift days and off-shift days and total scores on alcohol use were generated for each shift period. A score of ≥ 4 among men *(sensitivity 0.86, specificity 0.89)* and ≥ 3 among women *(sensitivity 0.73, specificity 0.91)* were classified as risky alcohol drinking (Bradley et al. 2007).

#### Smoking

Smoking status was assessed using 3 items. Participants were asked *“Do you smoke?”* and *“Have you ever smoked?”* (*Yes, No*). Participants were then classified as current smokers, ex-smokers or never smoked. Current smokers were then asked to report the number of cigarettes typically smoked per day, separately, during on-and off-shift days.

#### Physical activity

The *International Physical Activity Questionnaire (IPAQ)-short form* (International Physical Activity Questionnaire 1998) was used to measure participants’ physical activity during on-and off-shift days. The IPAQ measures the frequency (in days) and duration (in minutes) of mild, moderate-and vigorous-intensity physical activities that lasted for at least continuous 10 min in the last 7 days. In this study, we assessed physical activities during leisure time, and separate questions were asked for on-shift days and off-shift days. Moderate physical activities were indicated as activities making one breath to some extent tougher than usual (e.g., *lifting lighter weights, biking at moderate speed, or playing tennis in pairs*) whereas vigorous activities were activities making one breathe considerable tougher than usual (e.g., *lifting heavy weights or strenuous exercises*). Mild physical activities included walking (International Physical Activity Questionnaire 1998).

The weekly metabolic equivalent minutes (MET minutes) were computed for the various activities by multiplying the minutes and days by their established intensity (in METs): *walking* = *3.3, moderate* = *4.0 and vigorous* = *8.0 METs* (International Physical Activity Questionnaire 2004). The overall weekly physical activity was then estimated by adding mild, moderate and vigorous MET minutes. Using the criteria *“5 or more days of any combination of walking, moderate-intensity or vigorous-intensity activities achieving a minimum of at least 600 MET-min/week”* (International Physical Activity Questionnaire 2004)*,* which has been deemed sufficient physical activity for health benefits (Kyu et al. 2016)*,* participants’ physical activity measures were classified into insufficient physical activity: scores < 600 MET-min/week and sufficient to high physical activity: scores of > 600 MET-min/week (Biernat et al. 2008). The test–retest reliability for the IPAQ-short form scale is indicated high (α < 0.80) (International Physical Activity Questionnaire 1998).

#### Fruits and vegetable intake

Fruits and vegetable intake during on-and off-shift days were assessed using the items: “*How many serves of vegetables do you usually eat each day during on-shift days?”* and *“How many serves of fruit do you usually eat each day during on-shift days?”* and scored on an 8 point response scale *(1 serve, 2 serves, 3 serves, 4 serves, 5 serves, 6 serves or more, 7 less than one serve, 8 Don’t eat fruit/vegetables)* (Australian Bureau of Statistics 2011). Using the Australian daily dietary guidelines on minimum daily recommended serves of 2 or more fruits combined with 5 or more serves of vegetables, participants’ intake was classified as either adequate or inadequate intake by whether they usually do (adequate) or do not (inadequate) achieve these guidelines. (Australian Bureau of Statistics 2018a).

#### Overweight and obesity

Participants self-reported their height in meters and weight in kgs. Body Mass Index (BMI) was calculated and categorised as *underweight (BMI* < *18.5), normal weight (18.5–24.9), overweight (25–29.9) and obese (BMI* ≥ *30)* (Australian Institute of Health and Welfare 2020).

### Exploratory variables

#### Socio-demographic characteristics

The socio-demographic characteristics collected included participants’ age, sex, ethnic background, relationship status and duration, number of children, and highest educational level.

#### FIFO work-related measures

Work-related measures included current occupational role (management, administrative, services, professional, maintenance, technician, production, drilling, construction, labourer, machinery operator and driver, catering and other), and duration of working in FIFO. Others were regarding their work schedules, including the usual FIFO shift pattern (regular fixed day, regular fixed night and rotating shift), the usual number of hours of their normal shift (length), the typical number of consecutive days away at work, and the typical number of consecutive days at home.

### Data analysis

STATA version 13 software *(StataCorp LP, College Station, Texas, USA)* was used to analyse the data. Continuous variables were presented in means and standard deviations and the categorical variables in frequencies and proportions for descriptive purposes. The paired t-test and McNemer’s analysis were done, where appropriate, to examine the difference in sleep outcomes, alcohol consumption, smoking, physical activity, weight/obesity and fruits and vegetable intake over workers’ on-and off-shift days. Univariate and multiple logistic regressions (adjusting for socio-demographic, health behaviours and FIFO work-related factors) were conducted at a p < 0.05 statistical significance level to examine the socio-demographic, health behaviours and FIFO work-related predictors of physical health status and psychological distress of FIFO workers. The existence of multi-collinearity in the model was checked using the tolerance test by estimating the Variance Inflation Factor (VIF) value and the results, (VIF ranging from 1.13 to 1.88) showed no multi-collinearity.

## Results

### Background characteristics of the study participants

The characteristics of the participants are outlined in Table 1. Of the 299 participants who consented to take part in the study, 216 fully completed the survey (giving a completion rate of 72.2%) and were included in the analysis. The mean age of the participants was 39.9 ± 11.6 years, with more than half (62.0%) aged above 34 years. The majority of the participants were males (66.2%) and of Caucasian/White ethnic background (84.7%). About one-third of the participants had primary/secondary education or equivalent (32.4%). More of the participants were married (43.0%) or in De-facto/co-habiting /Civil partnership (25.5%) and more than half had at least 1 child (57.9%).

**Table 1:** Distribution of demographics and work-related characteristics of FIFO workers (*N* = 216)

Personal characteristics	Frequency	Percent
Age in years		
≤ 24	12	5.6
25–34	70	32.4
35–44	67	31.0
45 +	67	31.0
Sex		
Male	143	66.2
Female	73	33.8
Ethnicity		
Caucasian/White	183	84.7
Other	33	15.3
Relationship status		
Single/never married	43	19.9
Married	93	43.0
Separated/Divorced/Widowed	25	11.6
De-facto/co-habiting/Civil partnership/Other	55	25.5
Number of children		
None	91	42.1
1	27	12.5
2	51	23.6
3 +	47	21.8
Educational status		
Primary/Secondary education and equivalent	70	32.4
Trade/Apprentice	45	20.8
TAFE/College	60	27.8
Bachelor degree	30	13.9
Postgraduate degree	11	5.1
FIFO role		
Management Administration/services	54	25.0
Professional	27	12.5
Maintenance/Technician	39	18.1
Production/Drilling/Construction/Labourer	45	20.8
Machinery operator and driver	35	16.2
Catering/Other	16	7.4
Shift pattern		
Rotation shift (mix of day/night shift)	121	56.0
Regular shift (fixed day/fixed night)	92	42.6
Other	3	1.4
Shift hours		
< 12 h	30	13.9
12 h	129	59.7
> 12 h	57	26.4
Consecutive days spent at work		
< 8 days	43	19.9
8–14 days	156	72.2
15 + days	17	7.9
Consecutive days spent at home		
< 8 days	187	86.6
8–14 days	29	13.4
FIFO duration		
< 5 yrs	87	40.3
≥ 5 yrs	129	59.7

Most of the participants have worked in FIFO work arrangements for 5 years or more (59.7%), spending 8 days or more (80.1%) at the worksite and less than 8 days at home (86.6%) in a FIFO roster cycle. Furthermore, most of the participants indicated working on rotation shifts of a mix of day and night shifts (56.0%) or regular fixed day shifts (42.6%), and most of them reported working 12 h per day or more (86.1%). One-fourth of the participants (25.0%) reported working in management/administrative/services roles (see Table 1).

## Health-related behaviours of FIFO workers during on-and off-shift FIFO work periods

### Sleep duration and quality

During on-shift days, the participants self-reported shorter sleep duration (6.3 ± 1.2 h) compared to off-shift days (7.5 ± 1.5 h) (*p* < 0.001). More of the participants reported sleeping for 7 or more hours during off-shift days compared to on-shift days (78.2% vs 46.3%, *p* < 0.001). About 2 in 5 of the participants (40.3%) self-reported poorer sleep quality during on-shift days compared to 15.7% who self-reported poorer sleep quality on off-shift days (*p* < 0.001).

### Smoking and alcohol intake

About 1 in 4 of the participants (26.4%) were current smokers and 29.2% were ex-smokers. The average number of cigarettes smoked per day was similar during on-shift days and off-shift days (11.7 ± 6.9 vs 11.2 ± 7.5, *p* = 0.718).

The majority of participants (86.1%) reported to consume alcohol. More consumed alcohol at risky levels (AUDIT-C scores ≥ 3 for females and ≥ 4 for males) during off-shift days compared to on-shift days (57.9% vs 34.3%, *p* < 0.001).

### Fruits and vegetable intake

During off-shift days, the participants self-reported consuming more serves of vegetables per day (2.8 ± 1.4 serves) compared to 2.3 ± 1.3 serves during on-shift days (*p* < 0.001). A higher proportion reported consuming the recommended 5 or more serves per day during off-shift days compared to on-shift days (9.3% vs 4.2%, *p* = 0.013). However, the amount of fruits intake reported was similar during on-shift and off-shift days (1.7 ± 1.2 vs 1.7 ± 1.3 serves per day, *p* = 0.583); with similar proportions of FIFO workers consuming 2 or more serves per day for the period of the on-and off-shift days (52.8% vs 48.6%, *p* = 0.262).

### Physical activity and Body Mass Index

The study participants reported more MET minutes of mild/moderate/vigorous physical activities per week during on-shift days than on off-shift days (3531.6, SD = 4973.2 vs 2762.6, SD = 3385.2, *p* = 0.018). However, the same proportion of FIFO workers was found to engage in sufficient to high mild/moderate/vigorous physical activity per day during the on-and off-shift days (73.1% vs 74.5%, *p* = 0.755).

The study participants reported an overall average body mass index (BMI) of 28.4 ± 5.9 kg/m^2^ and 71.4% of them were classified as overweight or obese.

### Physical health and psychological distress

The majority of participants were classified as having good physical health status (91.2%). About one-third of the study participants (33.4%) reported a high to very high risk of psychological distress. The distribution of the physical health and risk of psychological distress is presented in Table 2

**Table 2:** Health status and related behaviours of FIFO workers (*N* = 216)

Health and related behaviours	On-shift days, m ± sd/n (%)	Off-shift days, m ± sd/n (%)	*p*-value
Sleep duration	6.3 ± 1.2 h	7.5 ± 1.5 h	< 0.001*
< 7 h	116(53.7)	47(21.8)	** < 0.001**^**¥**^
7 + h	100(46.3)	169(78.2)	
Sleep quality			** < 0.001**^**¥**^
Fairly good/very good	129(59.7)	182(84.3)	
Fairly bad/very bad	87(40.3)	34(15.7)	
Alcohol intake			
Non-risky	142(65.7)	91(42.1)	** < 0.001**^**¥**^
Risky	74(34.3)	125(57.9)	
Smoking			
Non-smokers	96(44.4)		
Ex-smokers	63(29.2)		
Current smokers	57(26.4)		
Number of cigarettes smoked per day	11.7 ± 6.9	11.2 ± 7.5	0.592*
Vegetables intake per day	2.3 ± 1.3	2.8 ± 1.4	< 0.001*
< 5 serves	207(95.8)	196(90.7)	**0.013**^¥^
5 + serves	9(4.2)	20(9.3)	
Fruits intake per day	1.7 ± 1.2	1.7 ± 1.3	0.583*
< 2 serves	102(47.2)	111(51.4)	0.262^¥^
2 + serves	114(52.8)	105(48.6)	
Mild/moderate/vigorous physical activities (MET-mins/week)	3531.6 ± 4973.2	2762.6 ± 3385.2	0.018*
Insufficient	58(26.9)	55(25.5)	0.755^¥^
Sufficient to high	158(73.1)	161(74.5)	
Body Mass Index			
Underweight	7(3.2)		
Normal/health weight	55(25.4)		
Overweight	77(35.7)		
Obese	77(35.7)		
Physical health status			
Poor	19(8.8)		
Good	197(91.2)		
Psychological distress			
Low risk	85(39.3)		
Moderate risk	59(27.3)		
High risk	44(20.4)		
Very high risk	28(13.0)		

*p-value from paired *t* test

^¥^Exact McNemar significance probability; Bolden significant at *p* < 0.05

### Personal and FIFO work-related characteristic associations with physical health and psychological distress

The results of univariate and multiple variable logistic regression are outlined in Table 3. At the univariate level, the results showed FIFO workers aged over 44 years compared to those less than 35 years (OR 0.49, 95% CI 0.25–0.99) were at reduced odds of psychological distress. Higher odds of distress were evident in workers who had no children compared to workers with children (OR 2.09, 95% CI 1.18–3.72), were current smokers compared to those who did not smoke (OR 3.64, 95% CI 1.82–7.28), and those whose shifts lasted for 12 h or more compared to those on shift less than 12 h (OR 2.82, 95% CI 1.03–7.70).

**Table 3:** Univariate and multiple logistic regression models of demographic and work-related characteristics predicting high/very high psychological distress and physical health (*N* = 216)

FIFO work characteristics	High/very high psychological distress		Poor physical health	
	Unadjusted OR (95% CI)	Adjusted OR (95% CI)	Unadjusted OR (95% CI)	Adjusted OR (95% CI)
Age in years				
< 35	1	1	1	1
35–44	0.53(0.27–2.30)	0.53(0.20–1.40)	1.02(0.30–3.51)	2.44(0.38–15.53)
> 44	0.49(0.25–0.99)*	0.37(0.13–1.06)	2.22(0.76–6.47)	4.99(0.76–32.71)
Sex				
Male	1	1	1	1
Female	1.17(0.64–2.11)	0.83(0.35–1.94)	1.54(0.62–3.83)	2.53(0.60–10.59)
Ethnicity				
Caucasian/White	1	1	1	1
Other	0.60(0.25–1.40)	0.66(0.22–1.99)	0.56(0.12–2.51)	0.18(0.02–1.61)
Relationship status				
Married	1	1	1	1
Single/never married	1.76(0.83–3.74)	1.15(0.36–3.70)	0.40(0.08–1.93)	0.24(0.03–1.85)
Separated/Divorced/Widowed	0.61(0.21–1.79)	0.41(0.11–1.52)	1.13(0.29–4.47)	0.58(0.09–3.60)
De-facto/co-habiting /Civil partnership/Other	1.63(0.81–3.29)	1.04(0.38–2.84)	1.02(0.35–2.97)	0.81(0.19–3.54)
Have children				
Yes	1	1	1	1
No	2.09(1.18–3.72)*	1.84(0.70–4.81)	0.83(0.33–2.09)	1.84(0.43–7.82)
Educational status				
Primary/Secondary education or equivalent	1	1	1	1
Trade/Apprentice	1.09(0.50–2.37)	1.06(0.40–2.81)	0.93(0.21–4.09)	0.98(0.16–6.11)
TAFE/College	0.90(0.44–1.86)	0.70(0.27–1.83)	2.60(0.84–8.09)	3.35(0.82–13.72)
Bachelor/postgraduate degree	0.58(0.24–1.38)	0.55(0.16–1.86)	1.03(0.23–4.54)	1.29(0.15–10.96)
Alcohol intake				
No	1	1	1	1
Yes	1.00(0.44–2.27)	0.57(0.19–1.68)	0.96(0.27–3.50)	1.09(0.21–5.59)
Smoking status				
No smoker	1	1	1	1
Ex-smoker	0.89(0.42–1.86)	1.34(0.54–3.31)	2.28 (0.69–7.52)	2.05(0.42–9.98)
Current smoker	3.64(1.82–7.28)***	7.17(2.67–19.26)***	3.41(1.08–10.75)*	5.65(1.13–28.32)*
Physical activity				
Adequate	1	1	1	1
Inadequate	1.79(0.96–3.33)	1.92(0.86–4.32)	0.42(0.12–1.50)	0.19(0.04–0.99)*
Body Mass Index				
Normal weight	1	1	1	1
Underweight	1.42(0.29–7.02)	0.92(0.11–7.98)	2.89 (0.26–32.35)	5.33(0.28–10.78)
Overweight	0.86(0.41–1.79)	1.24(0.50–3.05)	2.29(0.59–8.90)	3.07(0.50–18.97)
Obese	0.97(0.47–2.00)	1.45(0.58–3.63)	2.01(0.51–7.95)	2.51(0.42–15.05)
FIFO role				
Management/Administration/services	1	1	1	1
Professional	1.33(0.47–3.74)	1.37(0.38–5.00)	0.38(0.04–3.40)	1.50(0.09–24.70)
Maintenance/Technician	1.58(0.63–3.93)	1.63(0.53–5.04)	1.12(0.28–4.47)	3.47(0.55–22.04)
Production/Drilling/Construction/Labourer	2.10(0.89–4.98)	1.53(0.54–4.38)	1.82(0.53–6.14)	2.05(0.44–9.61)
Machinery operator and driver	2.37(0.95–5.91)	0.73(0.22–2.45)	0.59(0.11–3.25)	0.66(0.06–6.84)
Catering/Other	1.43(0.42–4.89)	1.57(0.35–7.05)	1.40(0.24–8.01)	0.82(0.09–7.47)
Shift pattern				
Rotation shift/others	1	1	1	1
Regular shift	0.61(0.34–1.10)	0.76(0.34–1.67)	1.25(0.51–3.09)	1.59(0.46–5.55)
Shift hours				
< 12 h	1	1	1	1
≥ 12 h	2.82(1.03–7.70)*	6.63(1.84–23.91)**	0.35(0.12–0.99)*	0.18(0.04–0.75)*
Consecutive days spent at work				
8–14 days	1	1	1	1
< 8 days	0.94(0.46–1.93)	0.72(0.29–1.78)	1.04(0.32–3.34)	1.21(0.27–5.39)
15 + days	0.81(0.27–2.42)	1.60(0.36–7.11)	2.17(0.55–8.49)	3.29(0.53–20.57)
Consecutive days spent at home				
< 8 days	1	1	1	1
8–14 days	0.48(0.19–1.23)	0.48(0.16–1.49)	0.65(0.14–2.97)	0.54(0.08–3.60)
FIFO duration				
≥ 5 yrs	1	1	1	1
< 5 yrs	1.19(0.67–2.11)	0.89(0.41–1.94)	0.90(0.36–2.28)	1.33(0.32–5.52)

*p < 0.05; **p < 0.01; ***p < 0.001

Reference category: used largest category for age, sex, ethnicity, marital status, education, have children, job, shift pattern, days spent at work and at home, and FIFO duration; and normative category for alcohol, smoking, physical activity, BMI and shift hours)

However, the odds of poor physical health status were lower in FIFO workers whose shift lasted for 12 h or more compared to those on shifts less than 12 h (OR 0.35, 95%CI 0.12–0.99). The odds of poor physical health status were higher in current smokers compared to those who did not smoke (OR 3.41, 95%CI 1.08–10.75).

In the multiple logistic regressions adjusting for socio-demographic characteristics (age, sex, ethnicity, marital status), health behaviours and work-related factors, the odds of high psychological distress were higher in those whose shifts lasted for 12 h or more compared to those on shifts less than 12 h (OR 6.63, 95% CI 1.84–23.91) and in workers who were current smokers compared to those who did not smoke (OR 7.17, 95% CI 2.67–19.26). On the odds of poor physical health, FIFO workers whose shift lasted for 12 h compared to those on shifts less than 12 h (OR 0.18, 95% CI 0.04–0.75) and reported inadequate physical activity (OR 0.19, 95% CI 0.04–0.99) had lower odds, whereas current smokers had higher odds compared to non-smokers (OR 5.65, 95% CI 1.13–28.32) (see Table 3).

## Discussion

The FIFO lifestyle of working on-and off-shift periods presents workers with two different contexts, characterised by different roles and lifestyles, which demand the taking on of diverse social roles and behaviours (Gardner et al. 2018). Our study investigated the self-reported mental and physical health and health-related behaviours of FIFO workers during on-and off-shift days.

We found longer self-reported sleep duration and better sleep quality during off-shift days than on on-shift days. Several studies have also made similar findings of shorter sleep duration during on-shift days than during off-shift days in FIFO workers (Paech et al. 2010; Tuck et al. 2013). Specifically, sleep duration reported during on-shift days (6.3 ± 1.2 h) was lower than the recommended 7 or more hours of sleep (Hirshkowitz et al. 2015). This aligns with the observations made by previous studies, which reported between 5.7 and 6.7 h of night sleep during work periods (Ferguson et al. 2010; Paech et al. 2010). Furthermore, the study found FIFO workers report better sleep quality during off-shift days than on on-shift days. Consistent findings have been documented in previous studies (Rebar et al. 2018; Tuck et al. 2013).

Sleep problems in rotation (FIFO) workers in the resources sector have been highlighted in a review (Asare et al. 2021a). FIFO workers typically work long hours (mostly 12 h) and day or night shifts and swing shifts (a mix of day and night shifts) patterns (Storey 2010), with early start times for day shifts. Working long hours (Rhéaume and Mullen 2018) and shift patterns, particularly night and early morning shifts (Kecklund and Axelsson 2016) and swing shifts, which require mid-roster shift changes (from day to night or night to day) (Parkes, 2016) have been linked with sleep disorders due to disruptions to the circadian rhythm (Sachdeva and Goldstein 2020). Early start times for day shifts require that workers sleep early to be up early and ready to catch a bus for work, but early evening hours bedtime is deemed frustrating and does not certainly result in early sleep onsets, as such the early start times to shifts may truncate sleep periods (Ferguson et al. 2010). Our findings suggest FIFO workers may accumulate sleep debt during on-shift days whereas there is an indication of recovery during off-shift days. FIFO campsite or village accommodations are designed to create sleep conditions such as a quiet environment (Sibbel et al. 2016) and limited room lighting during daytimes, but competing personal and social activities during non-work times at campsites are deemed to also interfere with sleep (Ferguson et al. 2010) and may need to be limited to promote sleep at worksites. Additionally, adjusting early start times to shifts (Ferguson et al. 2010) and allowing enough days off (between consecutive rosters) and longer changeover widow among workers on swing shifts, possibly greater than 24 h or days could allow for sufficient sleep recovery (Paech et al. 2010). For instance, a qualitative study has suggested that FIFO workers perceived a reduction in fatigue and a positive impact on their general health and well-being when a roster pattern of 7 day-shift/7 night-shift/7 days off changed to 10 day-shift/5 days off/8 night-shift/5 days off, and the latter to 8 day-shift/6 days off/8 night-shift/6 days off (Devine et al. 2008). Enabling adequate sleep could help address the negative effects that may accompany accumulated sleep loss, such as fatigue and impaired performance and related work accidents that can occur as a result of poor sleep or lack of sleep.

Our study showed that 26.4% of the FIFO workers self-reported as current smokers. This is comparable to the findings previously published among FIFO workers (Joyce et al. 2013) and the general mining workers (James et al. 2020) in Australia. These rates are higher than the 11.6% reported among adults in Australia (Australian Institute of Health and Welfare 2021b). FIFO workers work under stressful conditions such as extended separations from family, working long periods and on compressed shifts with increased work demands/workload (Langdon et al. 2016; Vojnovic et al. 2014a, b) and report severe distress levels (Bowers et al. 2018; James et al. 2018; Vojnovic and Bahn 2015). Increased stress levels are connected with the urge to smoke and more cigarette smoking (Stubbs et al. 2017), and could account for more of the FIFO workers engaged in smoking. Stressors associated with FIFO work may be profound during work periods, and as found in a previous daily diary study, FIFO workers smoked more cigarettes during on-shift days than on off-shift days (Rebar et al. 2018). In contrast, our study found FIFO workers smoked a similar average number of cigarettes per day during on-shift and off-shift days and the number of cigarettes smoked per day during on-shift days and off-shift days in our study was comparable to the 12.9 cigarettes per day reported in the general smoking population in Australia (Australian Institute of Health and Welfare 2021b).

Consistent with the findings reported in previous studies (Clifford 2009; Parker et al. 2018), a high proportion of the FIFO workers were found to consume alcohol. The rates of self-reported alcohol intake in the FIFO workers (86%) are higher than the reported rate (77%) of alcohol intake in the last year in the broad Australian populace (Australian Institute of Health and Welfare 2021a). Again, consistent with other previous studies (Clifford 2009; Joyce et al. 2013), the current study found more FIFO workers consume alcohol at risky levels during on-and off-shift days. Alcohol intake at higher levels among FIFO/rotation workers (Asare et al. 2021a), and in the general mining population in Australia (James et al. 2021; Tynan et al. 2017) have been previously documented. Studies have documented FIFO workers consume alcohol at higher levels than the general population (Dittman et al. 2016) and other workgroups (Joyce et al. 2013).

FIFO work population are largely men (Vojnovic et al. 2014a) and, with men known to typically consume more alcohol (Barker and Taylor 2019), this could account for the higher levels of alcohol consumption in FIFO workers. Additionally, FIFO work characteristics such as working rotating shifts and shifts length of more than 12 h, and the experience of high levels of psychological distress among the mining work population have been linked to risky/harmful levels of alcohol consumption (James et al. 2021). FIFO workers experience high levels of psychological distress (Bowers et al. 2018; James et al. 2018) as they face increased emotional demands, for instance, dealing with the ‘physical and psychological distance’, loneliness and isolation due to their absence from families (Gardner et al. 2018), which could also account for the high level of alcohol consumption in this workgroup. The presence of *‘wet mess’* at campsites and workplace culture which support social drinking (Carter et al. 2008; Torkington et al. 2011), is indicated to foster drinking at risky levels (Henry et al. 2013). However, in line with previous studies (Rebar et al. 2018; Tuck et al. 2013), our study found a higher proportion of FIFO workers consumed alcohol at risky levels during off-shift days than on-shift days. The level of alcohol consumption during on-shift days may be related to some level or full alcohol restrictions at some worksites (Tuck et al. 2013) and also the common requirements at almost all worksites for pre-shift breath tests for alcohol and unplanned substances/drugs testing before being allowed to work to ensure workplace safety (Australian Mines and Metals Association 2016). Such mandatory requirements do not extend to non-work times or off-duty at the campsites, where in places with no or some restrictions workers could engage in risky levels of alcohol intake (Duncan 2009). But, recent industry guidelines require companies to implement campsites measures including limiting the number of drinks taken in a 24-h window and serving varied strength of alcohol options including 0% alcohol strength beverages to ensure psychological and physical safety (The Chamber of Minerals & Energy of Western Australia 2022). Whereas at home, there may be more alcohol available and drinking among workers is seen as a sign of freedom from the worksite restriction (Collinson 1998). Experiencing boredom during off-shift days is also indicated to foster drinking (Carter et al. 2008). Interventions and strategies, including stress management (Chiesa and Serretti 2014), limiting the availability and access to alcohol on-site during shift days, for example, through restrictions on sales or replacing with non-alcoholic beverages and promoting awareness of the harmful effects of drinking and smoking (World Health Organization 2018) could help to address the relatively high levels of smoking and alcohol intake among the FIFO workers.

The self-reported daily intake of fruits and vegetables among FIFO workers in the current study was low. However, a comparable proportion of FIFO workers achieved the nationally recommended intake for fruits (52.8% on on-shift days and 48.6% on off-shift days) to that reported in the general adult (51.3%) Australian population (Australian Bureau of Statistics 2018a). More FIFO workers met the daily requirement for vegetable intake during off-shift periods (9.3%) than is typical for the general Australian population (7.5%) (Australian Bureau of Statistics 2018a). A similar study has also reported a high proportion of FIFO workers to consume insufficient fruits and vegetables but was not different from other workgroups in Australia (Joyce et al. 2013). FIFO workers in our study consumed more vegetables during off-shift days than during on-shifts. A daily diary study also found FIFO workers perceived their nutritional intake during off-shift days to be of a higher quality than during on-shift days (Rebar et al. 2018). FIFO workers potentially engage in more unhealthy eating behaviour (consuming food of poorer nutritional quality) (Asare et al. 2021a; Gibson et al. 2015), and that has been attributed to the readily accessible food that may be unhealthy at workplaces (Riethmeister et al. 2016). However, we did not assess unhealthy intake. High stress can be associated with unhealthy eating behaviours (Hill et al. 2021), including a high intake of food high in calories (Tryon et al. 2013) and less intake of foods low in calories such as fruits and vegetables (Mikolajczyk et al. 2009). With FIFO workers engaged in stressful work (Gardner et al. 2018; Torkington et al. 2011) and indicated to experience distress at high levels (Bowers et al. 2018; James et al. 2018), this could also explain the low intake of fruits and vegetables among the workers, particularly when on-shift.

Furthermore, more than half of the participating FIFO workers (71.4%) were classified as overweight or obese based on self-reported height and weight, somewhat higher than the proportion of the broad adult Australian populace (67%) reported as overweight or obese (Australian Institute of Health and Welfare 2020). Our finding compares favourably with those reported in the literature, which shows that FIFO workers have a higher prevalence of overweight and obesity than reported in groups working non-FIFO schedules in Australia (Joyce et al. 2013) and the universal population (Asare et al. 2021a). There could be fewer healthy food options available at work sites (Riethmeister et al. 2016; Sibbel et al. 2016) or less priority on healthy food choices among workers. For instance, Sibbel et al. (2016) have found FIFO workers to be less satisfied with the variety of food options and healthy food options available at campsites. Also, the potential of stress-induced eating due to high distress (Asare et al. 2021a) seen in FIFO workers is known to contribute to overweight and obesity (Paans et al. 2018). Organizations should provide more healthy food options at worksites (Leedo et al. 2017), educate workers to choose healthy food options and assist workers to cope better with the stressors inherent within the FIFO ‘lifestyle’ and reduce stressors wherever possible. This may help to improve the eating behaviours of workers and reduce the associated risks including non-communicable diseases and reduce productivity losses (World Health Organization 2021).

Our study found that most FIFO workers engage in sufficient to high self-reported mild/moderate/vigorous physical activities per week. This reflects the findings from the extant literature, which suggests high levels of leisure-time physical activities among rotation workers (Asare et al. 2021a). Furthermore, FIFO workers were found to engage in more MET-mins/week on on-shift days, but similar proportions of workers engaged in sufficient to high physical activities during both on-and off-shift days. This contradicts the finding of a previous daily diary study where FIFO workers reported less exercise time during on-shift days than off-shift days (Rebar et al. 2018). The differences between the measurements and designs used in our study (using one-off measures) and the previous study (using daily measures, which are less prone to recall errors) should be noted. The presence and accessibility of recreational facilities (Perring et al. 2014; Sibbel et al. 2016) such as gyms or wellness centres with health and well-being officers, basketball and tennis courts, swimming pools, and the organisation of sporting and other recreational activities including basketball, soccer and group fitness activities at worksites may have encouraged workers to engage in physical activities during on-shift days (Perring et al. 2014). Further, taking part in recreational activities on-site is indicated to promote social interactions and a sense of belonging (Perring et al. 2014) that support workers to manage separations from their families (Gardner et al. 2018). The closeness of camps limits the commute to and from worksites, allowing for some free time, and on-site recreational facilities are maintained and promoted, which encourages physical activities (Perring et al. 2014).

The higher level of self-reported physical activities observed in the current study could reflect the high proportion of FIFO workers (91.2%) being classified as having good physical health status. This finding is in the line with the extant evidence, where FIFO workers indicate having good to very good overall physical health status (Asare et al. 2021a), and the use of medications for physical health impairments as unusual (Rebar et al. 2018).

However, the study shows that 33.4% of FIFO workers reported a high to very high risk of psychological distress higher than reported in the Australian population (13.0%) (Australian Bureau of Statistics 2018b). Similar previous studies have also reported a higher prevalence of high to very high psychological distress among FIFO workers than documented in the Australian general population (Bowers et al. 2018; Lester et al. 2015; Sellenger and Oosthuizen 2017). FIFO work arrangements mean workers may be absent from their families for a long period and may not be able to carry out their family duties and miss some important family events. In line with the work-family theory (Allen et al. 2000; Greenhaus and Beutell 1985), the demands of being absent from home interfering with the accomplishment of family duties could result in stress-related outcomes at levels dependent on the significance of the unaccomplished task (Greenhaus and Beutell 1985). It has been highlighted that the difficulties of balancing work and home life (Gardner et al. 2018), not being able to attend to family emergencies and missing out on important family events (Gardner et al. 2018), and worries about maintaining family and social relationships (Gardner et al. 2018; Torkington et al. 2011) are all potential sources of distress for FIFO workers. Again, FIFO work comes with high demands of long/compressed roster patterns and shift hours (typical of 12 h), FIFO roles/workload, living away from families and dealing with loneliness and social isolation (emotional demands), and work-home interference (Vojnovic et al. 2014a), which have been highlighted in the extant literature to be associated with psychological distress among FIFO workers (Asare et al. 2021a). Furthermore, workers travel long distances between worksites and home during their days off, which may take off some time spent at home (reducing the available time for recovery and to spend with families), particularly among drive-in drive-out (DIDO) workers and those who FIFO into the city but need to drive to their home far in remote locations, which can potentially add to workers’ distress levels (Parker et al. 2018; Henry et al. 2013). In line with the Job Demands- Resources Model (Bakker and Demerouti 2007), high job demands contribute to strain including psychological distress. Current regulations in Australia require FIFO organizations to provide support for workers experiencing distress (Commission for Occupational Safety and Health 2019). There are on-site support programs and services (Ebert & Strehlow 2017; Torkington et al. 2011) such as counselling services (Ebert & Strehlow 2017). However, it has been indicated that some workers are reluctant to seek mental health support (Gardner et al. 2018; Torkington et al. 2011) for fear of losing their jobs, being regarded as weak (display of ‘macho’ culture) and the lack of awareness of when to seek help (Gardner et al. 2018). On-site support programs, such as on-site chaplaincy, have been suggested in a qualitative study to help workers overcome the fear of losing their jobs and workplace culture of masculinity associated with seeking help for mental health issues promoting the mental health and well-being of workers, with service provision related to *active outreach, trust-building, availability and confidentially* thought to be crucial in its effectiveness (Ebert and Strehlow 2017).

In the current study, FIFO workers who worked on shifts 12 h or more were at increased risk of experiencing high to very high psychological distress. A similar finding has been documented among mining workers in Australia (James et al. 2018). Again, smoking could be propagated by increased stress (Stubbs et al. 2017) and was associated with the health status of workers in this study. Specifically, workers who were current smokers had a high risk of experiencing high to very high psychological distress and poorer physical health status compared to workers who were not current smokers. This finding aligns with that of a previous study among mining workers in Australia, where workers who were daily smokers were more likely to experience a high level of psychological distress (Considine et al. 2017). Other personal and work-related factors including gender, employment type, length of FIFO experience and shift patterns were found not be associated with physical health and psychological distress, similar to the findings noted in a previous study (James et al. 2018). Contrary to previous studies in the mining industry (Bowers et al. 2018; James et al. 2018, 2020), factors including age, marital status, educational status, alcohol use and days spent away at work and at home (roster length) were not associated with physical health and psychological distress in adjusted models. The differences in the sample sizes of the studies, where previous studies involved larger sample sizes compared to our study, should be noted. Our findings suggest the experience of poor physical health and psychological distress may be widespread across the different groups of the study sample. However, the demands of work-related factors are indicated to be significant determinants of the health of workers (Asare et al. 2021a; Vojnovic et al. 2014a), and such factors have the potential to be modified within the work settings and could be the focus for interventions aimed at improving the health and well-being of FIFO workers (James et al. 2018; Considine et al. 2017). As such, studies with larger sample sizes and longitudinal designs may be needed to further examine the short and long-term impact of personal and work-related factors on health outcomes in FIFO workers.

The current COVID-19 pandemic and accompanying social/travel restrictions have been found to negatively influence health-related behaviours (Neill et al. 2020; Stanton et al. 2020) including smoking, alcohol intake, physical activity and sleep (Stanton et al. 2020) and given rise to an upsurge in psychological distress (Fisher et al. 2020) in the general population. COVID-19 restrictions, border closures and quarantines across the states in Australia prompted prolonged FIFO rosters and restricted workers travelling back home and thus, further prolonging the separations and isolation of FIFO workers from their families and friends. Again, COVID-19 restrictions and quarantines/self-isolation on-sites may limit socialization and other social activities on sites, causing further isolation and loneliness (Gilbert et al. 2020). Although the mental well-being levels of FIFO workers were found to be within normal limits during the pandemic (Asare et al. 2021b), it has been documented that prolonged rosters, restrictions on travelling back home and prolonged separations from home and families necessitated by COVID-19 restrictions were concerns for high levels of psychological distress reported among FIFO workers (Gilbert et al. 2020). Increases in stress caused by COVID-19 and its associated restrictive measures could have fostered increases in risky health-related behaviours (Neill et al. 2020). During this study (data collected between July and December 2021), there were still COVID-19 restrictions, border closures and quarantines in place in Australia. This could also have contributed to the high prevalence of psychological distress and risky lifestyle behaviours reported in FIFO workers in this study.

## Strength and limitations of the study

Our study contributes to evidence on the physical and mental health and their work-related predictors and has provided an overview of the health-related behaviours of FIFO workers during on-and off-shift periods. The study employed multiple recruitment sources and validated scales to present “snapshot” differences in the health-related behaviours across the two distinct components of the FIFO lifestyle—on-shift and off-shift periods—which could then be compared to the population norm data. However, recruiting from multiple sources has the potential to present a diverse sample (possibly from different organizations) with differences in work arrangements and practices, which may need further study to explore the impact of such differences on the health and well-being of FIFO workers. The use of a cross-sectional design excludes any causal interpretation of findings. The non-random sampling technique to recruit participants may be an appropriate approach to recruiting from an unstable population such as FIFO workers is also acknowledged as a limitation. However, our sample profile reflects that of a previous large study, where the FIFO work population is mostly males, middle-aged, married/in a form relationship, with TAFE/college education, and spent 8 days at work (Parker et al. 2018). Due to a larger rate of incompletion than expected, our analytic sample was below the number we set out to achieve for estimating the rate of distress with the desired precision. However, the analytic sample was sufficient to test small on-shift versus off-shift differences, which was the primary aim of this study. Online surveys are indicated to have high dropout rates, where not being able to meet the researcher (anonymity) may lead to a higher risk of dropout (Dandurand et al. 2008). It should be noted that, besides the negative impact of COVID-19 on the lives of individuals, the pandemic has also brought several changes in work arrangements including lockdowns and restricted access to mining sites, further exacerbating the already existing problems of limited chance to meet workers, which is characteristic of research at mining sites (Bowers et al. 2018). However, recruitment was also done through the social media platform Facebook to recruit general FIFO mining workers in Australia. Additionally, while this study explored the difference in health behaviours during on-and off-shift days, further studies could consider examining the influence of workers’ personal and work-related characteristics such as sex, age, and FIFO roles on their health behaviours across the FIFO roster cycle. Data on whether FIFO workers were engaged as full-time employees or as contractors were not collected. However, contractors compared to full-time employees may be treated differently by operating companies, such as having fewer statuary protections including sick or annual leave, and compensation, and may face arbitrary dismissal without redress. With contractor employment on the rise, we suggest further studies could explore the differences in the health and well-being in these two FIFO work groups.

## Conclusion

FIFO workers participating in this study reported good physical health status but higher levels of psychological distress compared to the Australian normative data. Our results further highlighted more of the FIFO workers were overweight or obese, smoked more, drink more alcohol at risky levels than in the general Australian population, and consumed fewer fruits and vegetables as compared to recommended guidelines. More of the FIFO workers also engaged in sufficient physical activity. The study also indicated that, during on-shift periods, FIFO workers had shorter sleep duration and poorer sleep quality, lower consumption of vegetables, and higher levels of alcohol consumption, but spent spend more MET minutes per week in physical activities. No substantial differences in smoking or fruit intake between on-and off-shift days of the FIFO roster cycle were found. The study identified working on long shifts of 12 or more hours and being a smoker to be associated with high to very high psychological distress. Interventions should attempt to alleviate psychological distress and support multiple health behaviour changes among FIFO workers. Such interventions could consider FIFO work-related characteristics including long shift hours. Additional studies exploring how behaviour change interventions could positively influence health and well-being of FIFO workers are required. Furthermore, longitudinal research is also warranted to investigate how day-to-day variations in psychological and contextual variables change over time.

## Data Availability

The data that support the findings of this study are not publicly available due to ethical and privacy restrictions, but available on request from the corresponding author on reasonable request.
